# Primary cervical meningeal melanocytoma with a dumbbell shape: Case report and review of the literature

**DOI:** 10.1097/MD.0000000000033435

**Published:** 2022-04-07

**Authors:** Weina Hou, Jianmin Yu, Sumei Gao, Yujing Chu

**Affiliations:** a Department of Radiology, Weifang People’s Hospital, Weifang, Shandong, China; b Department of Pathology, Weifang People’s Hospital, Weifang, Shandong, China.

**Keywords:** case report, magnetic resonance imaging, meningeal melanocytoma, spine, surgical therapy

## Abstract

Meningeal melanocytoma is a rare benign pigmented tumor originat from leptomeningeal melanocytes. Here, we report the case of a female who presented with numbness and weakness of the limbs for approximately 6 months.

**Patient concerns:**

We report the case of a 60-year-old Chinese female who presented with numbness and weakness of the limbs for approximately 6 months. computed tomography (CT) and magnetic resonance imaging (MRI) revealed a dumbbell-shaped tumor inside and outside the cervical (C) spinal canal.

**Diagnoses:**

The patient was using CT and MRI. Subsequently, the patient underwent surgery, and low-grade melanocytoma was diagnosed pathologically.

**Interventions:**

Subsequently, the patient underwent a surgery, and the tumor was completely removed.

**Outcomes:**

The tumor did not recur after 6 months.

**Conclusion:**

This case suggested 2 “take-away” lessons: first, spinal meningeal melanocytomas may be dumbbell-shaped; and second, melanocytoma could appear as hyperintense, isointense, or hypointense on T2-weighted MRI.

## 1. Introduction

Meningeal melanocytoma is a type of benign pigmented tumor that originates from leptomeningeal melanocytes. This tumor was first reported by Limas and Tio in 1972.^[1]^ Meningeal melanocytoma is an extremely rare, pigmented tumor of the central nervous system.^[2]^ Primary melanocytoma in the spinal canal is even rarer; 37 cases have been reported since 1972, and most articles were case reports. The tumor in the current case was a dumbbell-shaped cervical (C) intraspinal and extraspinal meningeal melanocytoma. The patient underwent comprehensive preoperative and postoperative imaging examinations, namely X-ray, computed tomography (CT), and magnetic resonance imaging (MRI). Typical tumors containing melanin granules demonstrate hyper intensity on T1-weighted imaging (T1WI) and hypo intensity on T2-weighted imaging (T2WI).^[3]^ However, our case did not show typical hypo intensity on T2WI. The clinical features, imaging findings, treatment options, and pathological features of this case are described in this article, with a comparison with the previous literature.

## 2. Case description

A 60-year-old Chinese female presented with numbness and weakness of the limbs. Numbness of the hands and feet had developed 6 months earlier, with no obvious cause and inducement, and gradually spread to the limbs, accompanied by tightness in the limbs and trunk. The patient denied a history of stroke, Parkinson disease, or other neuropathy. She initiated “traditional Chinese medicine” therapies on her own and applied a “plaster patch” for treatment, without improvement.

Physical examination showed that the patient had a poor C curvature. She reported tenderness in the spinous process and interspinous and paravertebral regions of the lower and upper C vertebrae, without obvious radiating pain or limited flexion and extension. Shallow hypesthesia was present in the skin of both upper limbs distal to the shoulder joint, and the skin in the pelvic region and both lower limbs distal to the groin. Shallow hypesthesia was not present in the skin of the trunk. No atrophy was seen in the patient limb muscles, and the muscle tension was high. The strength in bilateral deltoid, biceps brachii, triceps brachii, and the wrist flexor and extensor and hand intrinsic muscles was grade IV; the strength in bilateral iliopsoas, quadriceps femoris, tibialis anterior, gastrocnemius, and extensor dorsalis muscles was grade IV. Tendon and nerve reflex examinations showed bilateral biceps brachii tendon reflex: ++, radial periosteal reflex: ++, bilateral Hoffman sign: +, bilateral knee tendon reflex: +, and bilateral Achilles tendon reflex: +.

**Figure 1.**
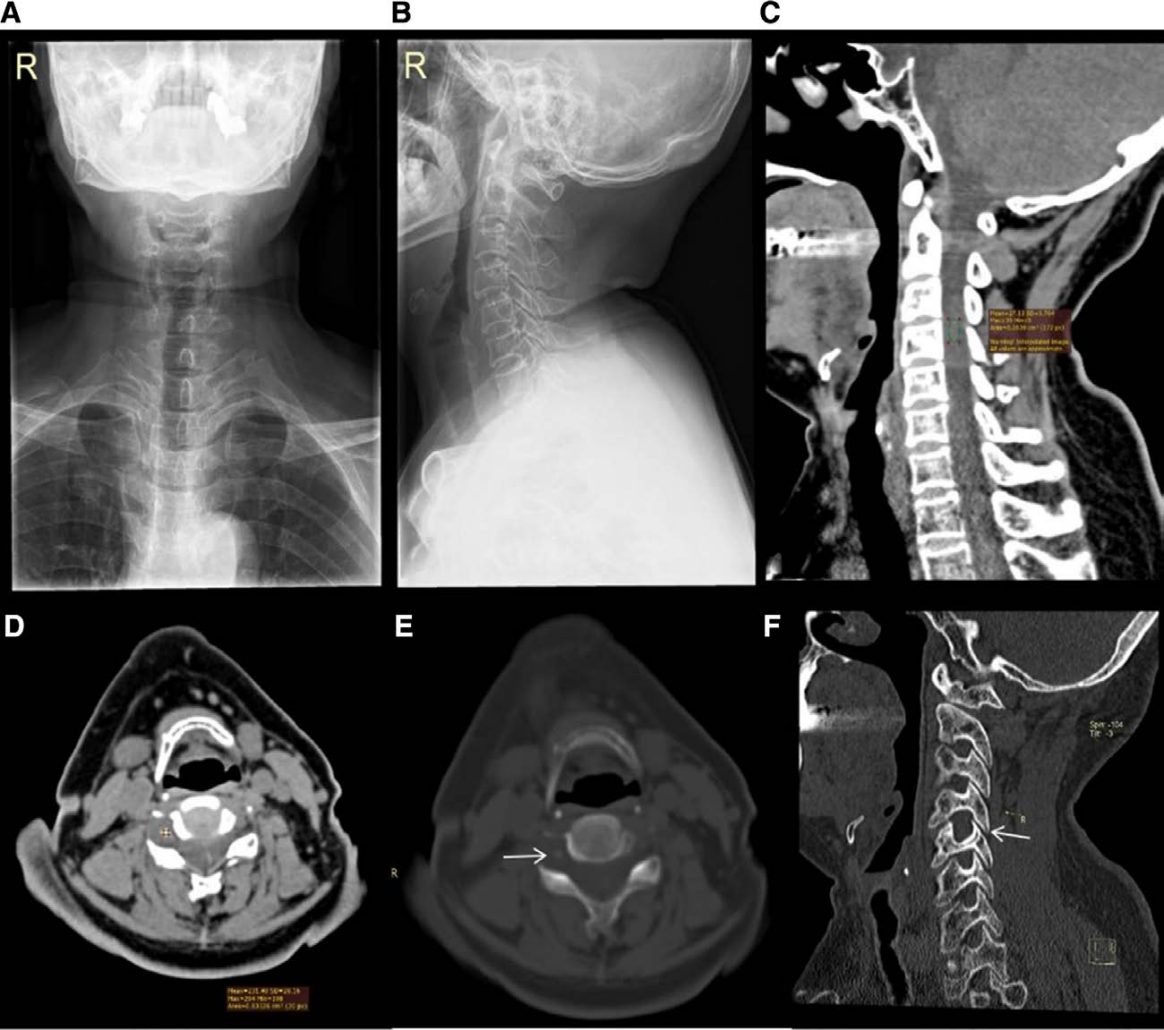
X-ray and CT of the cervical spine in a 60-yr-old female. Views: (A) X-rays in the posteroanterior and (B) lateral positions showing no obvious abnormalities. (C) CT sagittal soft tissue window showing that most of the tumor has a soft tissue density, with a CT value of approximately 27.13 HU. (D) CT axial soft tissue window showing irregular calcification in the tumor, with a CT value of approximately 231.40 HU. (E) CT axial bone window showing calcification (white arrow) in the tumor. (F) CT oblique sagittal bone window showing an enlarged intervertebral foramen at cervical 4/5 (white arrow). The adjacent bone is compressed and thin. CT = computed tomography.

**Figure 2.**
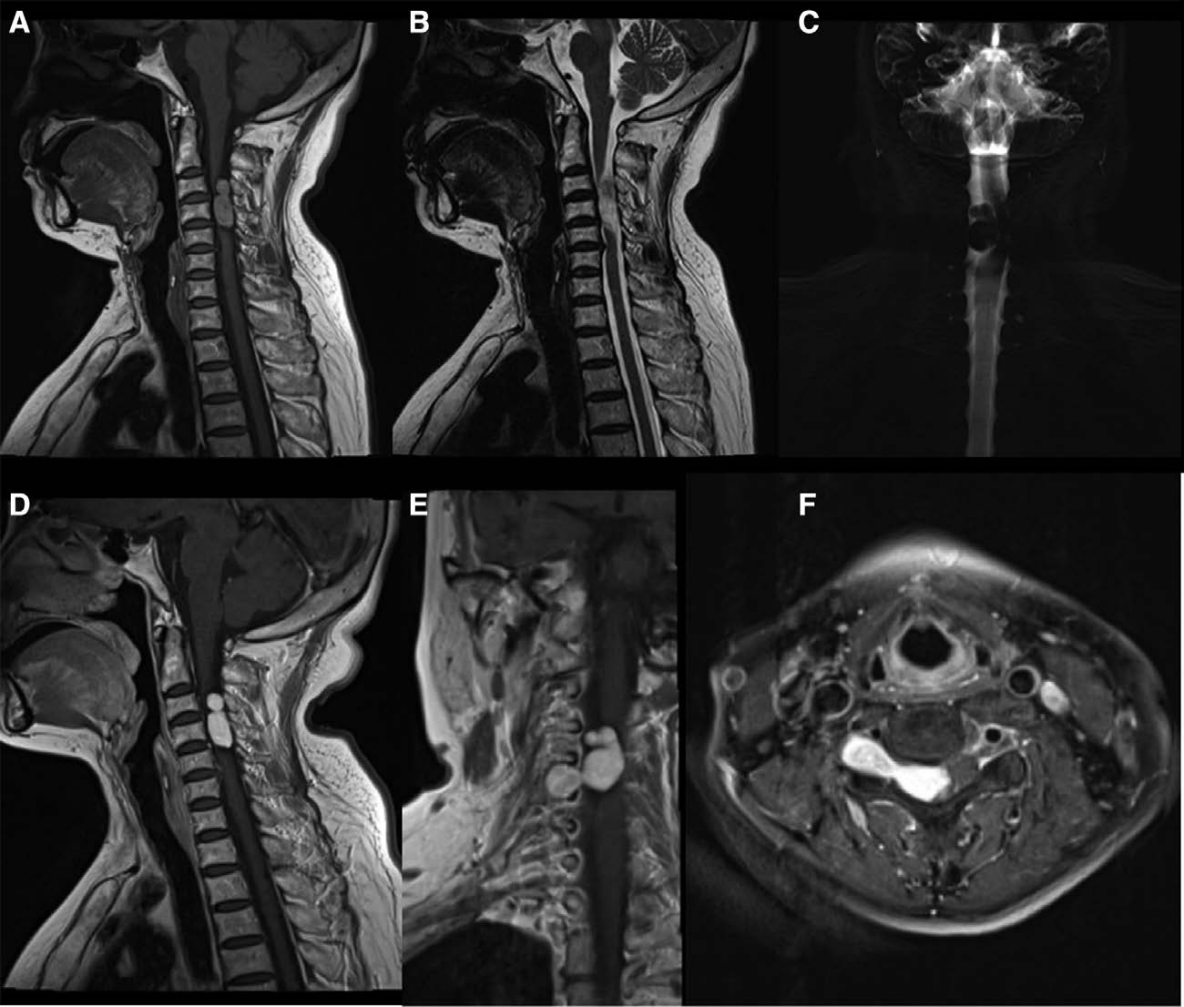
MRI of the cervical spine in a 60-yr-old female. Views: (A) The tumor was located at the level of cervical vertebrae 3 to 5 and appeared with high signal intensity on sagittal non-contrast T1WI. (B) The tumor showed uneven high signal intensity and a slight low signal intensity on sagittal T2WI. (C) Magnetic resonance myelography showed that the mass pushed the spinal cord to the left, and the subarachnoid space at the upper and lower edges of the mass widened, suggesting that the mass was located under the dura, not outside the dura. (D) The tumor is significantly enhanced, showing extremely high signal intensity on contrast-enhanced sagittal T1WI, although the tumor showed high signal intensity on non-contrast T1WI. (E) The tumor is dumbbell-shaped and located inside and outside the spinal canal, with the thinnest part located in the intervertebral foramen on coronal contrast-enhanced T1WI. (F) The tumor is dumbbell-shaped and hyperintense on contrast-enhanced axial fat-suppressed T1WI. MRI = magnetic resonance imaging, T1WI = T1-weighted imaging, T2WI = T2-weighted imaging.

The patient underwent X-ray, CT, and MRI with and without contrast. X-ray showed no obvious abnormalities (Fig. 1A and B). CT revealed a soft tissue density mass at the right side of the spinal canal at the level of the C3 to C5 vertebral bodies and the right intervertebral foramen of C4/5 (Fig. 1C–E). The intervertebral foramen of C4/5 was enlarged, and the adjacent bones were compressed and thin (Fig. 1F). The CT value of most of the mass was approximately 27.13 HU (Fig. 1C). Irregular calcification was seen in the mass, and the CT value was approximately 231.40 HU (Fig. 1D and E). MRI showed that the lesion was dumbbell-shaped and that the mass had grown across the intervertebral foramen. The size of the lesion was approximately 3.0 cm (transverse) × 2.8 cm (longitudinal) × 1.1 cm (anteroposterior). Non-contrast-enhanced MRI revealed high signal intensity in the tumor on T1WI and inhomogeneous high signal intensity on T2WI (Fig. 2A and B). MR myelography showed that the tumor had pushed the spinal cord to the left and widened the superior and inferior margins of the subarachnoid space, which indicated that the tumor was located in the subdural extramedullary space (Fig. 2C). The signal intensity of the tumor on MRI was significantly enhanced with contrast (Fig. 2D–F). MRI also showed disc herniations from C3 to C6.

The diagnosis on admission was a C intraspinal and extraspinal tumor with spinal cord compression. Two days after MRI, the patient underwent surgery for posterior tumor resection, internal fixation, bone grafting, and fusion. The surgeon removed the C3 to C5 spinous processes and lamina to expose the tumor. The tumor was located in the right posterior side of the canal at C3 to C4 and extended from the right foramina of C4/5 to outside the spinal canal, presenting as a dumbbell shape. The tumor had a complete capsule that was dark brown in color, with a rich blood supply and adhesions to the dura mater and C5 nerve root. The adhesions were separated, and the nerve root was protected. The tumor was completely removed and sent for pathological examination.

**Figure 3.**
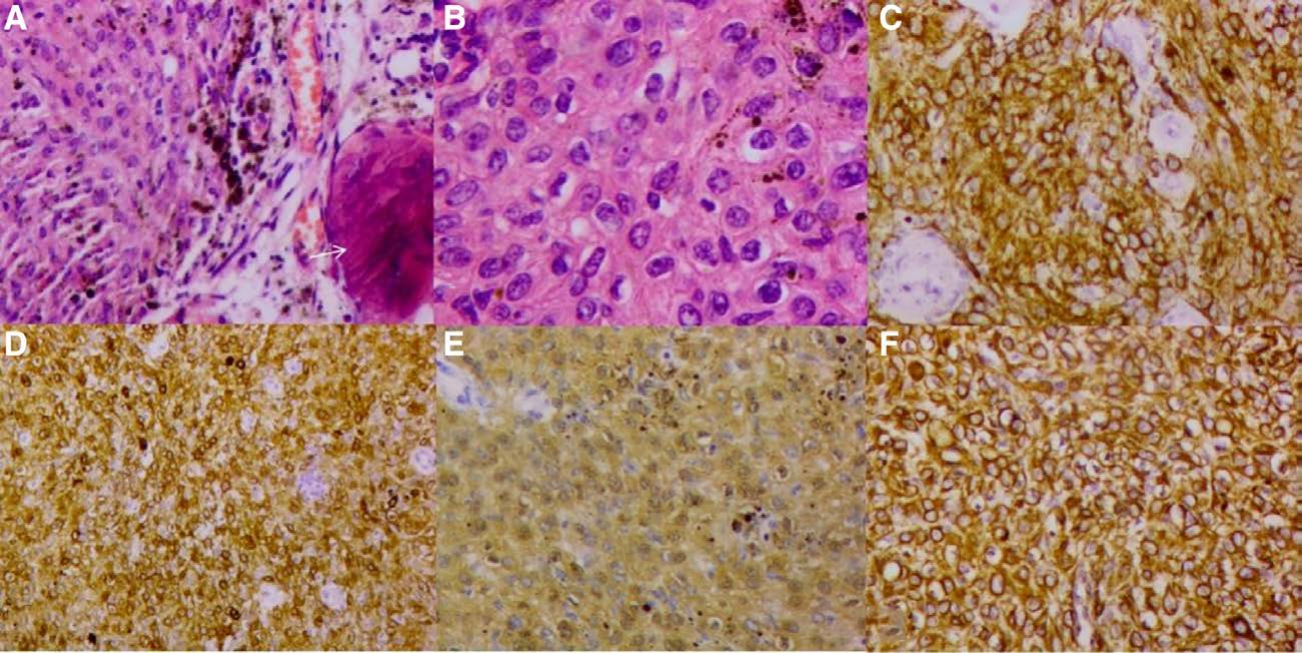
Pathological images. Views: (A) HE staining at × 20 magnification. Tumor cells are distributed in flakes and contain black melanin pigment and calcification (white arrow) in focal areas. (B) HE staining at × 40 magnification. The tumor cells show obvious nucleoli and focal pigment granules. (C) HMB-45 IHC staining at × 20 magnification. The cytoplasm stained diffusely positive. (D) Melan-A IHC staining at × 20 magnification. The cytoplasm stained diffusely positive. (E) S-100 IHC staining at × 20 magnification. The cytoplasm and nuclei stained positive. (F) Vimentin staining at × 20 magnification. The cytoplasm stained positive. HE = hematoxylin and eosin, HMB-45 = human melanoma black-45, IHC = immunohistochemistry.

The postoperative pathological gross specimen indicated grayish red irregular tissue with a complete tumor capsule. Most of the pathological section was grayish red and soft in texture, with some areas grayish-white in color and medium in texture. Microscopically, examination with hematoxylin and eosin stain showed melanocytic areas and consistent cell size. Mitotic figures were difficult to find (Fig. 3A and B). Immunohistochemical staining revealed the following: Human melanoma black-45 (+), Melan-A (+), vimentin (+), S-100 (+), pancytokeratin (individual +), P53 (individual +), cluster of differentiation 34 (partial +), epithelial membrane antigen (−), glial fibrillary acidic protein (−), and Ki-67 index (2%) (Fig. 3C–F). The final diagnosis was meningeal melanocytoma (low-grade tumor). The doctor thought that the patient would have a good outcome.

**Figure 4.**
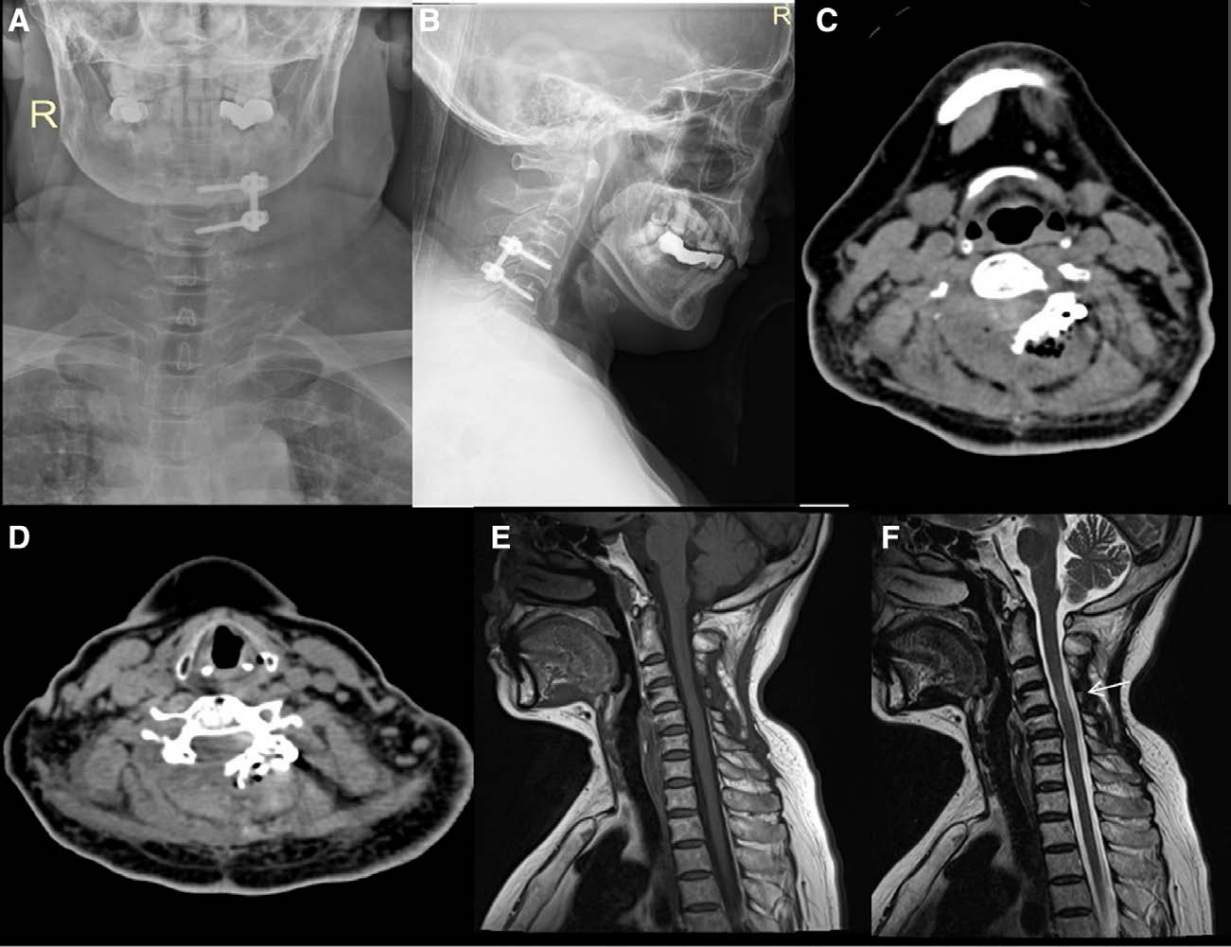
Postoperative images of the cervical spine of a 60-yr-old female. Views: (A) X-ray posteroanterior and (B) lateral positions showing that 1 screw has been inserted into the left cervical 4 and 5 pedicles, respectively, with stable internal fixation 2 wk after surgery. (C and D) Axial CT images showing no obvious bleeding in the operation area. A small amount of unabsorbed gas is visible 2 wk after the surgery. (E) Sagittal T1WI and (F) T2WI MRI showing no recurrence of the tumor 6 mo after surgery. A small amount of slightly high signal intensity is seen in the spinal cord (white arrow), suggesting there might be degeneration in the spinal cord. CT = computed tomography, T1WI = T1-weighted, T2WI = T2-weighted, MRI = magnetic resonance imaging.

The patient underwent X-ray and CT of the C spine 2 weeks after the surgery, which revealed normal postoperative change (Fig. 4A–D). The strength in the patient bilateral deltoid, biceps brachii, triceps brachii, wrist flexor and extensor, hand intrinsic, bilateral iliopsoas, quadriceps femoris, tibialis anterior, gastrocnemius, and extensor dorsalis muscles recovered to grade V. Six months after the surgery, the patient underwent repeat MRI examination, which showed no signs of tumor recurrence (Fig. 4E and F).

This case report was written in accordance with the CARE reporting guidelines 2013.

## 3. Discussion

Primary melanocytic neoplasms comprise diffuse leptomeningeal melanocytosis or melanomatosis, melanocytoma, and primary malignant melanoma.^[4]^ The incidence of primary central nervous system melanocytoma is estimated to be 1 per 10 million people.^[5]^ This case report demonstrated a rare C spinal meningeal melanocytoma located in the transforaminal region of the C spinal canal. The tumor appeared as an obvious dumbbell shape that was both hyperintense and hypointense on T2WI MRI. In the previous literature, we found only 4 cases of meningeal melanocytoma with a dumbbell shape and compared the findings in these cases with the findings in our case,^[6–9]^ which could improve the understanding of this disease. The limitation of our case report was that we did not obtain photos of the surgical process and gross pathology findings.

Table 1 summarizes the general clinical data of the 5 known cases of spinal meningeal melanocytoma with a dumbbell shape in intervertebral foramina, including the present case. We found that dumbbell-shaped spinal meningeal melanocytoma occurred mostly in women,^[6,8,9]^ and the proportion of women was higher than that in those with primary melanocytic neoplasms.^[4,10]^ We found that 4 cases (80%) of dumbbell-shaped spinal meningeal melanocytoma occurred in the C spine in our literature review.^[6,7,9]^ This might be related to swollen C nerves and the structure of the intervertebral foramen. Unfortunately, we found no evidence to support this theory, in this case. In our literature review, postoperative recurrence was reported in 3 cases of dumbbell-shaped spinal meningeal melanocytoma. The recurrence rate was higher than that in a study by Rahimi-Movaghar and Karimi.^[10]^ The reason might be that the dumbbell-shaped tumors were located both inside and outside the spinal canal in the recurrent cases, which made operation difficult and may have resulted in residual tumor or intraoperative dissemination.

**Table 1:** Clinical data in published cases of spinal meningeal melanocytoma showing a dumbbell shape (including the present case).

Author	Yearr	Age (yr)	Sex	Main clinical symptoms	Duration	Location	Side	Treatment	Outcome
El-Khashab et al.^[6]^	2009	17	F	Progressive tetraparesis	3 wk	C6/7	Left	Surgery	Moderate progression of residual tumor 14 mo later.
Miura et al.^[7]^	2019	40	M	Numbness of right palm and left leg	2 mo	C2/3	Right	Surgery	No recurrence 6 mo later.
Shaikh et al.^[8]^	2021	36	F	Low back pain	6 mo	L3/4	Left	Surgery	Recurred within 6 mo, it transformed malignant melanoma about 1 yr later.
Vaidya et al.^[9]^	2021	29	F	Postoperative quadriplegia	3 mo	C4/5	Right	Surgery+radiation	Recovered in 2 mo
Hou et al.	2022	60	F	Numbness and weakness of limbs	6 mo	C4/5	Right	Surgery	No recurrence 6 mo later

C = cervical, L = lumbar.

Table 2 summarizes the imaging findings in the 5 previous cases of spinal dumbbell-shaped meningeal melanocytoma. Calcification was seen in the melanocytomas, including in our case and that of El Khashab et al.^[6]^ Melanin particles contain a large number of free radicals and unpaired electrons, which can form chelates. These substances have paramagnetism, which can lead to the typical hyperintensity seen on T1WI and hypointensity on T2WI MRI of melanocytic neoplasms. Gaviani et al.^[11]^ reported that the content of melanin in the tumor was positively correlated with the signal intensity on T1WI: higher melanin content was associated with higher signal intensity on T1WI. However, the authors reported no correlation between melanin content and T2WI signal intensity. This could explain why our case showed hyperintense to hypointense signals on T2WI.

**Table 2:** Imaging findings in published cases of spinal meningeal melanocytoma with a dumbbell shape (including the present case).

Author	CT-calcification	Intervertebral foramen	MRI-T1WI	MRI-T2WI	Enhanced degree
El-Khashab et al.^[6]^	Yes	Enlarged	Hyperintense	Hyperintense	/
Miura et al.^[7]^	No	Enlarged	Hyperintense	Hypointense to isointense	Not visualized clearly
Vaidya et al.^[8]^	/	Enlarged	/	/	Homogeneously enhanced
Shaikh et al.^[9]^	/	Enlarged	Hyperintense	Hypointense	Homogeneously enhanced
Hou et al.	Yes	Enlarged	Hyperintense	Hyperintense to hypointense	Homogeneously enhanced

CT = computed tomography, MRI = magnetic resonance imaging, T1WI = T1-weighted imaging, T2WI = T2-weighted imaging.

Table 3 summarizes the ICH findings of the 5 previous cases of spinal dumbbell-shaped meningeal melanocytoma. In the current case, the positive expression of human melanoma black-45, S-100, and Melan-A diagnosed the tumor as a melanocytic neoplasm, which was consistent with previous studies.^[7–9]^ The difference between malignant melanoma and melanocytoma lies in the mitotic activity of cells, nuclear abnormalities, and the degree of melanin granule proliferation. According to the latest edition of the World Health Organization classification of central nervous system tumors, the Ki-67 index ranges from <1% to 2% in melanocytomas and is approximately 8% in primary melanomas.^[12]^ In this case, histopathologically, the tumor cells were relatively uniform in size, cells showing mitosis were difficult to find, and the Ki-67 index was 2%; therefore, the tumor in this case was diagnosed as a low-grade melanocytoma. The tumor was essentially benign and cured by resection alone.

**Table 3:** Immunohistochemical findings in published cases of spinal meningeal melanocytoma with a dumbbell shape (including the present case).

Author	HMB-45	S-100	Melan-A	Ki 67
El-Khashab et al.^[6]^	/	/	/	10%
Miura et al.^[7]^	(+)	(+)	(+)	1%
Vaidya et al.^[8]^	(+)	(+)	/	1%
Shaikh et al.^[9]^	(+)	/	/	3%-4%
Hou et al.	(+)	(+)	(+)	2%

HMB-45 = human melanoma black-45.

## 4. Conclusion

We reported a rare dumbbell-shaped spinal meningeal melanocytoma. Our case suggested 2 “take away” lessons: first, spinal meningeal melanocytomas may be dumbbell-shaped and located inside and outside the spinal canal, which would cause enlargement of the intervertebral foramen; second, melanocytoma always shows hyperintensity on T1WI MRI; however, hyperintensity, isointensity, and hypointensity all may be seen on T2WI MRI. The signal intensity of T2WI MRI is not characteristic of this disease.

## Author contributions

**Data curation:** Sumei Gao.

Investigation: Jianmin Yu.

Writing – original draft: Weilna Hou.

Writing – review & editing: Yujing Chu.
